# Supplementary material for: Are carbon emissions trading and green financial instruments synergistic? -Comprehensive quantitative research based on content analysis
Source: PLoS One. 2024 Mar 7;19(3):e0298601. doi: 10.1371/journal.pone.0298601 (PMC10919601; doi:10.1371/journal.pone.0298601)
Supplement: S1 Data — (ZIP) [file pone.0298601.s001.zip › Data/地级市Z_F工作报告.zip!/╒π╜¡/╬┬╓▌╩╨/└┤╫╘╣1⁄2╓┌║┼í╛┬φ┐╦╩2╛▌═°í┐_2016.docx]

各位代表：

　　现在，我代表市人民政府，向大会作政府工作报告，请予审议，并请市政协委员和其他列席人员提出意见。

　　一、2015年主要工作和“十二五”发展回顾

　　2015年是“十二五”规划的收官之年。面对严峻复杂的宏观形势和繁重艰巨的改革发展任务，我们认真贯彻落实省委、省政府和市委系列决策部署，实施“五化战略”，深化“十大举措”，克难攻坚、奋力拼搏，全力推进温州经济、城市和社会转型发展，较好地完成了市十二届人大五次会议确定的主要目标任务。

　　（一）保稳促调取得成效。积极推进产业集聚区、开发区（工业园区）整合提升，启动浙南科技城规划建设，开工建设市级示范小微园53个，瓯海时尚智造、苍南台商小镇等5个特色小镇列入省级创建名单。实施“五一〇”产业培育提升工程，制定出台领军企业、高成长型企业培育计划，积极推进“机器换人”和企业股份制改造，新增高新技术企业133家、“企转股”210家，技改投资增长24%。大力发展旅游、电商、物流等服务业，积极培育新的消费热点，社会消费品零售总额增长11%。现代农业发展势头良好，农业“两区”建设提质增效。深入实施“温商回归”工程，实际到位内资1110亿元。推出促进创新创业系列政策和“人才新政十条”，新入选“国千”6人、“省千”14人，首批8家市级众创空间挂牌。市县两级合力处置重点风险企业63家，处置不良贷款402亿元，不良贷款余额、不良率实现“双下降”，信贷投放企稳回升。全市生产总值4620亿元，增长8.3%，增速四年来首次高于全省平均水平；一般公共预算收入403亿元，增长7%；固定资产投资3456亿元，增长13.2%；城镇常住居民人均可支配收入44026元，农村常住居民人均可支配收入21235元，实际增长7.9%、8.7%。

　　（二）城乡建设步伐加快。强化都市区发展理念，调整完善城市总体规划和土地利用总体规划。全面启动新一轮市区城中村改造，完成整村连片改造签约3000户。滨江商务区、中央绿轴等城市亮点区块形象初现，三垟湿地整村搬迁进度加快，乐清、瑞安、泰顺、苍南等县（市）新区展现新面貌，一批市政、商贸、文教等配套基础设施建成投用。开展征地拆迁“清零”百日攻坚行动，市域铁路S1线、甬台温高速复线、绕城高速西南线等重点项目建设全面提速。金温铁路扩能改造工程、大门大桥建成投用，瓯江南口大桥、机场T2航站楼主体工程完工，市域铁路S2线、瓯江北口大桥、通用航空基地开工建设。完成围垦造地3万亩，沿江沿海标准堤塘实现闭合。实施城镇低效用地再开发2.6万亩，清理批而未供、供而未用土地5.6万亩。加强农村交通、环保、饮用水等基础设施建设，新建（改造）农村公路640公里，建成投用镇级污水处理厂16座，新增城镇一二级污水管网430公里，新增污水日处理能力24.4万吨。市综合材料生态处置中心试运行。

　　（三）环境整治有力推进。顺利通过国家历史文化名城、国家环保模范城市评估验收，成功创建国家园林城市，“六城联创”有望圆满收官。深化“五水共治”和“水岸同治”，实施河道综合整治186公里，完成1596个村生活污水治理任务。“三改一拆”“两路两侧”整治年度任务提前完成，拆除违法建筑1863万平方米，创建“无违建乡镇（街道）”26个。新建城市绿地349公顷，建成滨水公园220个；绿化造林10.9万亩。文成获得国家级生态县称号，洞头、泰顺入选首批国家级生态保护与建设示范区。创建美丽乡村精品线12条、精品村107个。实施大气污染防治“六大行动”，超额完成燃煤锅炉、黄标车淘汰任务，空气环境质量优良天数达312天。“一打三整治”取得阶段性成效。

　　（四）改革开放持续深化。启动实施金融综合改革“新十二条”，温州民商银行开业，温州资产管理公司完成注册登记，小微企业信用保证基金运行中心、小额贷款保证保险运营管理中心挂牌营业。以洞头撤县设区为契机，深化市区一体化发展研究。梳理市级功能区管理体制，完善乡镇（街道）行政区划。龙港国家新型城镇化综合试点启动，柳市入选国家首批中小城市综合改革试点。乐清、瑞安列入农民住房财产权抵押试点。平阳户籍制度改革试点取得新进展。分类推进市属国企改革，国资监管得到加强。获批海峡两岸（温州）民营经济创新发展示范区，设立温州韩国产业园。区域通关一体化稳步推进，口岸关检合作“三个一”改革取得初步成效，保税物流中心（B型）通过省级预验收。

　　（五）民生福祉不断改善。认真办好民生实事。改造义务教育薄弱学校57所，创建标准化学校182所，各县（市、区）全部通过国家义务教育发展基本均衡评估。县域辖区幼儿园实现“零无证”。创建省级以上中职学校30所、中高职一体化职业院校4所，一批高等院校建设步伐加快。深化公立医院改革，积极推进分级诊疗试点，市县两级门诊药费和住院药费实现“双下降”。出生人口性别比连续六年下降。温州文化艺术大楼和一批县级博物馆、文化中心建成投用，创新推出城市书房、文化驿站，建成农村文化礼堂208家。成功举办温州艺术节、市民文化节、中华龙舟大赛和首届温州马拉松赛。《温州两家人》掀起收视热潮。实施全民参保登记，完善大病保险制度，城镇和农村低保标准分别提高8%、12%。全面消除家庭人均年收入4600元以下贫困现象。深化十大重点领域安全生产专项整治，开展夏季消防安全“双月”攻坚行动，整治居住出租房及合用场所52.9万家，安全生产三项主要指标持续下降。妥善化解一批历史遗留问题和信访积案。严厉打击各类违法犯罪活动，社会治安形势持续向好。成功引入空中应急救援力量。“六五”普法圆满收官。工会、共青团、妇女儿童、老龄、慈善、红十字、残疾人等事业进一步发展，统计、档案、民族、宗教、双拥、援疆援藏、防台减灾等工作取得新成绩。

　　（六）政府自身建设进一步加强。深入推进“四单一网”建设，推广实施政府职能向社会组织转移“第五张清单”，非行政许可审批事项实现“清零”。启动行政审批制度重建改革，首创“网上开证明”系统。率先推行“五证合一、一照一码”，办证时限由20多个工作日压缩至3个工作日内。完善政务督查督办机制，去年《政府工作报告》提出的374项工作任务，完成或基本完成的有369项，完成率达98.6%。主动接受市人大法律监督、工作监督和市政协民主监督，办好人大代表建议497件、政协提案513件。获准行使地方立法权。认真开展“三严三实”专题教育活动，抓好中央“八项规定”落实和“四风”问题整改，正风肃纪常态化机制逐步形成。强化审计监督和监察问责，严肃查处一批违纪违法案件，反腐倡廉工作成效明显。

　　各位代表，2015年主要目标任务的顺利完成，标志着“十二五”规划全面收官。“十二五”规划纲要确定的29项主要指标，除地区生产总值、人均生产总值、消费、进出口、研发经费支出占生产总值比重、千人医生数、农村常住居民人均可支配收入等7项外，其他22项顺利完成预期目标。这五年，我们致力于控风险稳增长，经济实现企稳回升。地区生产总值年均增长7.8%，一般公共预算收入年均增长9.7%，主要经济指标在全省位次近两年明显前移；三次产业结构从3.2∶51.2∶45.6调整为2.7∶45.5∶51.8，实现了从“二三一”到“三二一”的转变。这五年，我们狠抓固定资产投资，城市和基础设施建设大幅推进。城市发展框架进一步拉开，交通、能源、水利、市政等网络加快形成，完成固定资产投资1.3万亿元，是“十一五”时期的3.8倍。这五年，我们大力推进“六城联创”，城乡环境面貌明显改观。累计拆违9772万平方米，新建了一大批城市公园，建成绿道3800多公里，人均公园绿地从6平方米提高到13平方米。这五年，我们持续推进改革攻坚，对外开放迈出新步伐。启动实施36个国家级和28个省级改革试点，金融综合改革、社会力量办学办医渐显成效，空港、海港口岸实现扩大开放。这五年，我们加快发展各项社会事业，民生保障水平稳步提高。城乡居民人均可支配收入年均实际增长7.1%、8.6%，一批教育、卫生、文体设施相继建成投用，养老、医疗等社会保障制度基本实现城乡全覆盖。

　　过去五年，我们在应对风险挑战中取得了显著成效，也在发展实践中积累了宝贵经验。一是必须遵循发展规律，善于从全国全省发展大局中找准定位、把握趋势，坚持从温州实际出发，科学合理设定目标，急而不躁、稳扎稳打地推进各项事业发展。二是必须坚持实体为基，积极有为搭平台、强要素、优环境，引导企业坚守实业、做强主业，防止“脱实向虚”，不断提升民营经济发展水平。三是必须持续改革创新，进一步解放思想、更新观念，勇于突破、敢闯新路，努力把改革开放这面旗帜举得更高，把温州的创新创业史写得更好。四是必须坚持依法行政，以法治政府建设的实际成效，引导全社会强化法治意识、规则意识和契约精神，提升诚信建设水平，着力构建经济社会发展的良好秩序。

　　各位代表，过去五年的发展成绩来之不易。这是省委、省政府和市委正确领导的结果，是全市人民齐心协力、奋力拼搏的结果。在此，我代表市人民政府向全市人民和外来建设者，向人大代表、政协委员，各民主党派、工商联、无党派人士和人民团体，向驻温部队、武警官兵和中央、省驻温单位，向情系家乡的海内外温州人，向所有关心和支持温州发展的港澳台同胞、海外侨胞和国际友人，表示崇高的敬意和衷心的感谢！

　　我们也清醒地看到，我市经济社会发展还面临不少困难、问题和挑战。主要表现是经济发展面临稳增长与调结构的双重压力，“两链三险”尚未根本解决，企业商务成本相对较高、生产经营依然困难，平台建设进展缓慢，创新要素支撑不足，新旧动力转换接续不畅；都市区统筹发展能力不强，城市规划建设管理水平有待提升，基础设施建设和公共配套相对滞后，“半城市化”问题突出，中心城区首位度不高；社会事业发展和社会管理任务艰巨，优质公共服务供给与人民群众的期望还有较大差距，社情舆情比较复杂，基层基础相对薄弱，维护社会和谐稳定的压力不小；政府自身建设有待加强，发展软环境存在不少问题，一些政府工作人员服务意识不强，不担当、不作为，“四风”问题和消极腐败现象仍然存在。对此，我们一定高度重视，并采取有效措施切实加以解决。

　　二、“十三五”时期的奋斗目标和主要任务

　　“十三五”时期，是新常态下推进温州转型发展、创新发展的关键期。根据中共温州市委《关于制定温州市国民经济和社会发展第十三个五年规划的建议》，市政府制定了《温州市国民经济和社会发展第十三个五年规划纲要（草案）》，提请大会审议。

　　按照市委十一届十次全会的部署，我市“十三五”发展的指导思想是：高举中国特色社会主义伟大旗帜，以马克思列宁主义、毛泽东思想、邓小平理论、“三个代表”重要思想、科学发展观为指导，深入贯彻习近平总书记系列重要讲话精神，以“四个全面”战略布局为统领，以创新、协调、绿色、开放、共享五大发展理念为指引，以“八八战略”为总纲，以“干在实处永无止境，走在前列要谋新篇”为新使命，以发展为第一要务，以提高发展质量和效益为中心，坚持和发展“温州模式”，深入实施“五化战略”，联动推进经济转型、城市转型、社会转型，努力建设民营经济创新发展示范城市、东南沿海重要中心城市、迈入全面小康社会标杆城市，为率先基本实现现代化奠定更加坚实的基础。

　　“十三五”发展的主要目标是：全市生产总值年均增长7%以上，到2020年达到6600亿元，人均生产总值达到72000元；城乡居民人均可支配收入分别达到65000元、32500元，实现“四翻番”；社会保障覆盖率达100%，研发经费支出占生产总值比重达到2.3%，使温州的综合实力更强、城乡区域发展更协调、生态环境更优美、人民生活更幸福、社会治理体系更完善。

　　今后五年，我们要围绕建设迈入全面小康社会标杆城市这一核心任务，重点在以下五个方面取得明显成效：

　　（一）打造民营经济创新发展示范城市。精准把握供给侧结构性改革的要求，以“中国制造2025”“互联网+”和创新创业为引领，深入实施“五一〇”产业培育提升工程，鼓励企业加快产业、技术、商业模式和治理结构创新，促进产业由中低端向中高端迈进。深入实施创新驱动发展战略，全力打造浙南科技城，构建国家高新区、国家经开区、产业集聚区、特色小镇、众创空间等多层次创新创业平台；大力培育高新技术企业，培养引进科技领军人才和创新团队，营造有利于创新创业的良好生态。全面实施“发现培育工程”，培育一批领军企业、高成长型企业，力争到2020年全市高新技术企业和科技型中小微企业数量翻一番。提升发展信息经济、金融服务业、商贸物流、生命健康等新型业态，第三产业增加值占生产总值比重达到55%。大力推进农业全产业链建设，打造一批农业产业集聚区和区域性农产品品牌。

　　（二）建设东南沿海重要中心城市。坚持以交通圈拓展都市圈，按照“直通高铁、加密高速、提升机场、优化港口、发展城轨、完善城网、建设枢纽”的要求，全力打造综合交通枢纽城市。按照“一主两副三极多点”的都市区空间布局，坚持市区一体化、瓯江两岸同城化、环大罗山协同化发展，完成市区70个以上城中村改造和三大亮点区块建设任务，中心城区首位度明显提升。谋划发展湾区经济带，打造产城融合的东部新增长极，实现城市从“瓯江时代”迈入“东海时代”。积极推动县域经济向城市经济转型，促进乐清、瑞安与市区相向融合发展，加快鳌江流域一体化，形成联系紧密、功能协调的城市发展新格局，常住人口城市化率达到70%左右。

　　（三）构建生态绿色美丽家园。巩固提升“六城联创”和美丽乡村创建成果，依托瓯越文化和山江海岛自然资源，统筹推进城乡建设与环境再造、生态保护与旅游开发，全力打造“美丽温州”。全面落实主体功能区规划，构建“三区三廊八核一带”生态安全新格局，努力成为全国生态文明先行区。深入开展城乡环境综合整治，强化水体、大气、土壤、近岸海域污染治理和生态修复，力争全面消除劣Ⅴ类水体，决不把违法建筑、污泥浊水、脏乱差的环境带入全面小康。深化重点行业和领域治理，确保完成“十三五”节能减排任务。健全生态环境保护制度，建设国家低碳试点城市，形成人与自然和谐发展的新局面。

　　（四）争当新一轮改革开放排头兵。坚持把为市场主体创造最优发展环境作为深化改革的出发点和落脚点，强化问题导向、结果评价，以更大的决心和勇气改革攻坚，续写温州改革开放新传奇。继续深化金融综合改革、农村综合改革、社会力量办学办医等国家和省级改革试点，加快各领域改革突破，形成一批可借鉴的改革样本和示范项目。切实转变外贸发展方式，大力发展跨境电子商务，积极培育进口商品市场，促进外贸“优进优出”。实施更加主动的开放带动与区域合作战略，鼓励企业走出去参与“一带一路”建设。主动融入长三角一体化发展，加快建设海峡两岸（温州）民营经济创新发展示范区，全面提升对外开放水平。

　　（五）让全市人民共享高水平的小康生活。顺应人民群众过上幸福生活的新期待，坚持普惠性、保基本、均等化、可持续方向，以“补短板”为重点，加快发展各项社会事业，提高优质公共产品供给能力，进一步增强人民群众的获得感。深入践行社会主义核心价值观，大力弘扬以创新创业为核心的温州人精神，深化“信用温州”建设，优化人文环境、提升全民素质。加强社会保障和救助体系建设，更加注重对特殊人群、困难群体的精准帮扶。积极应对人口老龄化，加快构建功能完善、规模适度、覆盖城乡的养老服务体系。深化平安温州建设，完善基层社会治理网络，健全公共安全和社会治安立体防控体系，确保社会和谐稳定。

　　三、2016年主要目标和重点任务

　　今年是“十三五”规划的开局之年，也是温州改革提速、发展提质、工作提效的关键之年。全市经济社会发展的主要预期目标是：地区生产总值增长7.5%-8%，一般公共预算收入增长7%左右，固定资产投资增长11%以上，城乡居民人均可支配收入实际增长7.5%，力争研发经费支出占生产总值比重达到2%，完成省下达的节能减排任务。重点做好以下七方面工作：

　　（一）坚持创新引领，着力增强发展内生动力。

　　全面掀起创新创业热潮。扎实推进浙南科技城建设，重点抓好基础设施、公共配套、互联网创新创业基地和人才社区建设，引进和集聚一批科研院所、企业研发中心，力争南起步区早出形象。支持在国家、省级开发区（工业园区）创建科技园，在有条件的高校设立创业学院，新增市级众创空间15家，力争龙湾众创社区当年建成投用。进一步深化产学研合作，积极引导社会资本参与创新投资，鼓励企业加大研发投入，组建一批产业技术创新联盟，新增高新技术企业150家，新增国家、省级企业技术中心和企业研发中心25家。加强新产业新业态用地政策创新，扩大创新券（服务券）应用。制定实施创业融资、居住保障、子女就学、贡献奖励等政策，切实优化人才发展环境，力争新入选“国千”5名、“省千”15名。实施温籍人才回归工程，支持“双元制”人才培养模式，培养高技能人才2万名。大力弘扬企业家精神，倡导开放包容的时代文化，营造鼓励创新、崇尚创业的浓厚氛围。

　　深化重点领域和关键环节改革。全面实施金融综合改革“新十二条”，争取开展区域资本市场区域中心建设试点，支持地方法人金融机构扩能升级，规范各类市场融资行为，构建完善区域金融稳定机制。实施资源要素差别化配置改革，全面推行正向激励与反向倒逼相结合的产业结构调整机制。积极推广公建民营、民办公助、混合经营等模式，促进国有和民办社会事业人才双向流动，推动民间资本顺利进入社会事业领域。启动市属国企新一轮整合重组，建立健全现代企业制度，加快温州银行、珊溪经济发展公司等优质国有资产上市步伐。深化商事制度改革，实行“一照一码”、全程电子化注册。开展行政审批标准化建设和公共服务便利化改革，推广在线服务、网上办理，建立重点区域、企业投资项目高效审批机制，争创政府职能向社会组织转移“第五张清单”全省示范。推进事业单位分类改革和机关事业单位养老保险制度改革。积极开展国家海域管理综合改革创新试点。深化农村综合改革，稳妥推进农村土地、产权和户籍制度改革，加快农村“三权”确权颁证，探索推进农村集体建设用地流转和宅基地利用管理创新。

　　进一步扩大对外开放与合作。加强跨境电商平台、外贸综合服务平台、国际贸易“单一窗口”建设，确保保税物流中心（B型）正式封关运作，积极申报温州综合保税区。加快发展水果、肉类等进口指定口岸业务，制定借鉴自贸区经验工作方案，争取市场采购贸易方式试点。稳步推进空港、海港客货运国际航线的开辟工作，力争乐清湾港区临时开放获批。依托海外温商网络资源，积极打造民外合作产业发展大平台，加快建设温州韩国产业园，切实提高利用外资的规模和水平。支持大企业到境外建立生产基地、营销网络，开展跨国并购重组。扎实推进海峡两岸（温州）民营经济创新发展示范区建设，积极申报台商投资区。

　　（二）坚持保稳促调，努力提高经济增长质量和效益。

　　推动产业向中高端提升。强化产业引领、创新驱动、产城融合理念，全力推进瓯江口产业集聚区、浙南产业集聚区建设，加快提升各类产业园区（平台）的引领示范和带动能力。支持乐清经济开发区申报国家级开发区。抓好30个省市级特色小镇创建和培育工作，集中精力打造若干个具有典型示范效应的特色小镇。加强小微园配套建设，确保竣工500万平方米。积极实施“五一〇”产业培育提升工程，启动“温州制造质量提升计划”“工业技改五年倍增计划”，抓好乐清电气、永嘉泵阀、瑞安汽摩配、鹿城鞋革等四大省级“机器换人”行业试点。主动顺应新一代信息技术发展趋势，积极引导轻工企业采用个性化设计、柔性化制造，推动传统优势产业进行产品全生命周期的再造。深化工业创强，全面实施领军企业、高成长型企业培育计划，大力推动现代企业制度建设和企业股份制改造，新增上市企业8家、新三板挂牌50家。深入实施“小微企业三年成长计划”，完成“个转企”1000家，新增规上企业300家、限上企业200家。加快淘汰落后产能，打赢重污染高耗能行业整治攻坚战。积极推进建筑工业化，支持建筑业做大做强。

　　促进服务业优质高效发展。制定实施服务业“双百”企业培育计划，争取省级服务业强县（市、区）试点。启动实施旅游发展三年行动计划，深化大旅游管理体制改革，加快推进雁楠旅游一体化，积极参与浙皖闽赣国家东部生态旅游实验区建设，支持洞头国家海洋公园、环飞云湖国家公园建设，谋划实施主题岛、风情小镇等特色精品旅游项目，打造“神奇山水·传奇温州”品牌。实施城市商圈提升三年行动计划，引进建设一批大型高端商业项目。大力发展楼宇经济，培育一批税收“亿元楼”。加快国际电工电器城、浙闽台水产城扩建等专业市场建设。结合东部综合交通枢纽建设，谋划发展区域性物流中心；继续推进空港新区物流园、乐清湾物流园区、瑞安江南物流园区等建设，支持第四方物流平台发展。着力提升会计、法律等现代服务业发展水平。科学把握供地结构和节奏，促进房地产业健康发展。积极培育时尚、电商等消费热点，争取网络零售额突破1200亿元，社会消费品零售总额增长10%。

　　做强做优现代农业。落实粮食安全责任制和最严格的耕地保护制度，完成永久基本农田划定，深化农业“两区”建设，确保20亿斤粮食生产能力。实施农业龙头企业培育计划，重点打造奶制品、铁皮石斛、花卉苗木、茶叶等一批农业特色产业，启动建设2个农业产业集聚区，形成一批现代农业强镇。深入实施“三位一体”合作组织示范创建行动，积极构建新型农业社会化服务体系。探索运用物联网等新技术推进农业生产精细化，培育提升一批农产品电商平台。加大金融支农力度，力争“整村授信”突破20亿元，新建市级扶贫资金互助会20家。积极培育现代林业经济。鼓励发展远洋捕捞、新型水产养殖业，加快建设渔港经济区和海洋生态牧场。认真做好第三次全国农业普查工作。

　　合力营造企业发展的良好环境。制定实施企业减负三年行动计划，落实各项税费减免政策，千方百计降低制度性交易成本、融资成本和工业用地成本。坚持分类化解企业“两链”风险，采取兼并重组、债务重组、破产清算等方式有效处置“僵尸企业”，严厉打击“逃废债”，确保不良贷款余额、不良率持续下降。加大金融服务实体经济工作力度，发挥好政府增信机制作用，争取制造业贷款增速高于全省平均水平，新增社会融资总量1100亿元。建设全市统一的信用信息交换平台，构建公开透明的社会信用环境。

　　（三）坚持重大项目带动，进一步扩大有效投资。

　　着力优化投资结构。启动实施重大项目建设三年行动计划，完成固定资产投资3840亿元，力争民间投资和工业投资占比分别达到64%、26%，重大基础设施项目、重大产业项目、生态保护和环境治理、高新技术产业投资均增长15%以上。量质并举推进招商选资，建立重大项目招商统筹机制，发挥产业园区、做地主体的主力军作用，重点瞄准新兴产业、行业龙头企业和创新型项目开展点对点精准招商，确保完成“温商回归三年超三千亿工程”。

　　加速构建现代基础设施网络。把交通建设摆在更加突出的位置，加快建设甬台温高速复线、绕城高速西南线和北线二期、龙丽温高速瑞文段，力争开工龙丽温高速文泰段。加快实施市域铁路S1和S2线、77省道延伸线、104国道西过境、228国道和330国道改扩建等市域道路骨架工程，抓好鳌江三座桥建设。完成杭温高铁预可，深化温武铁路、市域铁路S3线、金丽温高速东延线、龙丽温高速平苍连接线等项目前期。全力推进机场综合交通枢纽、通用航空基地、乐清湾港区铁路支线等空港海港基础设施建设。新建农村联网公路100公里、改造提升350公里，行政村通班车率达到93%。继续推进瓯飞一期、瓯江口新区二期、飞鳌中期等围垦工程建设，科学有序推进已围垦土地的开发利用。加快建设“天然气入温”工程，扎实推进浙江三澳核电、泰顺抽水蓄能电站项目前期。深入实施防洪减灾保安工程。加强城乡电网建设，新增变电容量307万千伏安，新增线路114公里。实施通信基础设施建设三年行动计划，推动通信基础网络扩容增效。

　　强化投资要素保障。深化投融资体制改革，充分利用政府和社会资本合作（PPP）、投资基金、专项债券等方式引导社会资本投资，组建市域轨道交通发展基金、基础设施财政引导基金，进一步拓宽政府性项目融资渠道。深化重点项目征地拆迁“清零”行动，依法打击影响项目建设的违法行为，切实净化投资发展环境。积极开展“坡地村镇”试点。加大批而未供、闲置土地清理力度，实施城镇低效用地再开发1.8万亩。建立市域用地指标统筹机制，确保重点建设用地指标需求。加强耕地占补平衡工作，完成滩涂垦造耕地1.5万亩、“旱改水”1万亩。

　　（四）坚持城乡区域统筹，加快都市区建设步伐。

　　完善都市区发展格局。深化温州都市区发展战略研究，注重从体制机制入手，加强与长三角、海西区和周边城市的对接合作。健全完善市域统筹协调、市区一体化发展机制，推动永嘉瓯北、三江片加快融入市区，推动乐清、瑞安与市区基础设施互联互通、产业优化布局，推动平苍副中心加快组团融合、提升城市功能。支持文成、泰顺建设国家生态主体功能区，以实施“小县大城、旅游小镇”为重点，促进生态功能板块后发崛起。切实抓好龙港国家新型城镇化综合改革试点、柳市国家级中小城市改革试点，积极培育一批新生中小城镇，构筑大中小城镇协调发展新格局。

　　加快城市建设步伐。科学制定年度做地、储备和出让计划，大力推进市区33个城中村整村连片改造，完成签约9000户。制定实施市区工业企业搬迁改造三年行动计划，分批连片推进老旧工业区改造提升。鹿城重点抓好滨江商务区、中央绿轴、双屿综合整治和七都新城等区块建设，积极培育环五马千亿级商圈，着力打造现代服务业与历史文化相融合的高品质核心区。龙湾重点抓好中心区、浙南科技城起步区、空港新区和状蒲片等板块建设，着力打造生产性服务业优势明显、创新要素集聚的东部发展中心。瓯海重点围绕中心区、站前商贸区、牛山片、梧白片等区块，抓好基础设施配套和重点项目招商，着力打造商贸、医教特色鲜明的高铁经济新城区。加强大罗山开发与管理，加快三垟湿地公园建设步伐。按照规划共绘、设施共建、服务共享的要求，洞头重点推进环海西湖城市核心区建设，启动全域景区化改造，加快同城发展，建设“海上花园”。以绕城高速交通圈、市域铁路网为纽带，制定实施沿线区块功能布局规划和开发建设计划。加强城市功能配套，加大学校、商贸、文体等公共服务设施“补缺”力度，提升城市宜居品质。

　　加强城市精细化管理。编制城市功能分区、城市设计导引、“城市家具”等规划方案，加快城市公共服务“一张网”建设，实现一张蓝图绘到底、一套规制管“四区”。实施城市道路建设和市政架空线路“上改下”三年行动计划。开展入城口整治和示范街（路）创建活动，市区重点打造8条示范街（路）。继续“打卡口、接断路”，集中整治交通堵点；制定实施停车场建设三年行动计划，强化乱停车、乱收费治理，着力缓解行车难、停车难问题。优化中心城区到乐清、瑞安、永嘉等周边地区公交线路。实施农贸市场改造提升专项行动。实施《温州市市容和环境卫生管理条例》，形成严管严治长效机制。推动城市管理重心下移，提高城市管养市场化、专业化水平。强化信息技术在城市管理中的应用，切实提高城市管理的智慧化水平。

　　（五）坚持生态优先，加快建设“美丽温州”。

　　强力推进环境综合治理。深化“五水共治”，扎实开展“清三河”与“水岸同治”，突出治理温瑞塘河及平原河网劣Ⅴ类水，河流交接断面水质达标率提高到80%。持续推进瓯江、飞云江、鳌江和重点中小流域综合治理，开工建设温瑞平原东片、西片排涝工程，加快瓯江标准海塘提升改造、江西垟平原防洪排涝、南岸水库、楠溪江河口大闸等项目前期。实施城镇污水处理厂提标改造三年行动计划，加大截污纳管力度，建设城镇一二级污水管网200公里，全面完成农村生活污水治理村建设任务。积极推进海绵城市建设。加强饮用水水源地保护，确保县级以上集中式饮用水源水质达标率100%。启动“温州湾”海洋生态修复工程。深入开展大气污染防治“六大行动”，全面启动省级以上工业园区集中供热设施建设，加快燃煤锅炉淘汰改造，统筹推进工业废气、建筑扬尘、餐饮油烟和废弃物焚烧治理。落实农业土壤污染防治三年行动计划。全面实施生活垃圾分类和减量化管理，建成永强垃圾发电厂二期、西向生态填埋场等项目。加大环保执法力度，严肃查处环境违法行为。完善全民参与机制，引导公众关注生态文明、参与环境保护。

　　提升“美丽温州”建设水平。加强城市公园绿化、行道树景观改造和山体修复，新建城市绿地570公顷，建设省级绿道140公里，绿化造林2.2万亩，城市主要道路彩化率达到25%。加大“三改一拆”“四边三化”工作力度，健全拆改结合、环境管护长效机制。深化“千村整治、百村示范”工作，制定实施美丽乡村建设行动计划，加强历史文化名镇名村保护，打造一批瓯派民居样板村，建设美丽乡村精品线10条、精品村100个，申报美丽宜居示范村20个。

　　健全“绿水青山就是金山银山”体制机制。深入实施西部生态产业带发展规划，鼓励发展生态旅游、养生养老等产业。制定实施民宿业发展扶持办法，推动农（渔）家乐休闲旅游提档升级。进一步完善生态补偿机制。严格实施污染物总量账户制度，推进刷卡排污系统建设、排污权有偿使用和交易。深化水、电、气等价格改革，促进资源节约集约利用。加快建设再生资源回收示范工程和16个循环经济试点基地，推广使用高效节能环保产品。

　　（六）坚持民生导向，提高公共服务供给水平。

　　努力办好群众满意的教育。深入开展教育现代化县（市、区）创建，加大教育投入力度，创建省义务教育标准化学校60所，培育“新优质学校”150所，努力打造“家门口的好学校”。深化市区两级教育管理体制改革，实施市区东部基础教育提升三年行动计划，促进教育均衡化发展。扎实推进第二轮学前教育三年行动计划，新建、扩建幼儿园60所，普惠性幼儿园覆盖面达到70%，等级幼儿园比例达到80%。继续抓好温州肯恩大学、浙江安防学院（筹）、浙江东方学院、温州医科大学仁济学院等一批高校校区建设。积极推动高等教育、职业教育办学教学模式改革，支持乐清、瑞安建设高职校区。鼓励民办教育优质特色发展，努力形成“公办保均衡、民办供选择”的教育新格局。

　　着力提高群众健康水平。坚持医疗、医保、医药联动，深化公立医院改革。推进分级诊疗和医生多点执业改革，实施全科医生培育计划和责任医生签约服务制度，促进优质医疗服务“双下沉、两提升”。建成市人民医院娄桥新院，加快建设附二医瑶溪新院和平阳、苍南县人民医院新院等医疗项目。强化疾病防控、卫生应急工作，抓好智慧卫生平台建设。积极扶持中医药事业发展。推进医院、养老机构双向改革，培育一批护理型示范养老机构，新增养老机构床位2000张、居家养老服务照料中心600家。有序实施“两孩政策”。深入开展全民健身活动，抓好国家体育产业联系点建设，向社会提供更多优质的体育服务。

　　切实增强城市文化实力。深入开展群众性精神文明创建活动，努力提高全民道德素质和社会文明程度。加强公共文化服务有效供给，做精城市书房、文化驿站，新建农村文化礼堂150家，办好第四届市民文化节。深入挖掘“瓯”系列文化，启动实施“斗城文化区”保护开发。实施农村文化骨干万人培育计划，建立非遗传承人保障培养机制，培养更多的民间文化能人、传承人和群众文化带头人。抓好温州国家广告产业试点园区建设，支持发展文创园、微电影等新型文化业态和特色文化产业项目。

　　加强就业和社会保障工作。坚持以创业带动高质量就业，重点做好高校毕业生和农村转移劳动力、就业困难人员等群体的就业服务工作。全面实施大病保险制度，实现医保智能监管平台全覆盖。逐步缩小城乡低保差距，农村低保标准达到城镇的85%。完善临时救助制度，实现救助对象全覆盖。积极发展慈善公益事业，支持有劳动能力的残疾人就业。做好城乡危旧房改造工作，扎实推进文泰震区安居工程建设。市区开工建设保障性安居房2万套，竣工1万套，交付8000套。组织实施精准扶贫计划，全面落实产业扶贫、挂钩帮扶等措施，完成异地搬迁1.5万人。加大对革命老区、少数民族地区、偏远山区和海岛地区的扶持力度。

　　完善为民办实事机制，重点办好缓解停车难、改善城乡出行、保障公共安全、强化餐桌安全治理、促进教育均衡化、提高医疗卫生水平、促进创业就业、优化养老助残服务、丰富文体生活、改善人居环境等十个方面实事，努力让人民群众得到更多实惠。

　　（七）坚持和谐发展，全力维护社会安全稳定。

　　加强平安温州建设。健全社会治安立体防控体系，推进社会治安物联网管控工作，严防严打各类违法犯罪和暴力恐怖活动，全力做好G20峰会等重大活动安保工作。加强社会矛盾排查预警，完善初信初访化解和人民调解机制，加大信访积案和历史遗留问题化解力度。深化“7431”网络治理模式，进一步提升“温州发布”“温州辟谣”平台的时效性和权威性。持续推进安全生产十大重点领域专项整治，深入开展以居住出租房、合用场所为重点的消防安全整治，强化特种设备、压力容器、涉氨涉尘作业场所等安全防范工作，加强交通运输安全管理，坚决遏制重特大事故。加强应急救援体系建设，提升快速反应能力。以更大力度抓好食品药品和农产品质量监管，确保“舌尖上的安全”。

　　强化基层基础建设。坚持把乡镇（街道）建设作为固本强基的关键环节，抓好行政区划调整后续工作；探索乡镇（街道）差别化管理，加强社会服务管理中心规范化建设，切实提升乡镇（街道）统筹协调、依法行政和公共服务能力。以村级组织换届为契机，加强村（居）民自治机制建设。实施村级集体经济三年培育计划，下决心解决一批“空壳村”问题。

　　加强和创新社会治理。深化社会组织登记制度改革，规范社会组织承接政府职能行为。分类推进城乡社区规范化建设，健全便民服务机制，着力构筑新型社区服务体系。优化社工人才培养使用和评价激励机制，新增持证社工1000人。改善外来人口服务与管理，完善以人才积分制为主体的居住证制度，推动市民卡、居住证、e居卡“三卡合一”。实施社会治理“一张网”工程，探索基于移动互联网、大数据、云计算、物联网等新一代信息技术的社会治理信息体系建设。推进军民融合深度发展，做好国防建设、国防教育、国防动员和双拥优抚安置等工作。积极支持工会、共青团、妇女儿童等事业发展。进一步抓好民族、宗教、外事侨务、对台、档案、地方志等工作。

　　四、加强政府自身改革和建设

　　牢固树立有为政府理念，以改革创新精神，全面加强和改进政府自身建设，努力打造敢于担当、执行有力、务实高效、勤政廉洁的政府。

　　坚持依法行政。加强法治政府建设，完善法治政府建设指标体系和考评制度。各级政府工作人员要学法、懂法、守法，积极运用法治思维和法治方式，正确行使职权，坚持法无授权不可为、法定职责必须为。全面推行综合行政执法。加强和改进行政复议工作，建立重大行政执法决定法制审核制度，切实规范行政自由裁量权，绝不能让权力任性而为。自觉接受市人大及其常委会的法律监督和工作监督，自觉接受市政协的民主监督，高质量做好建议、提案办理工作。认真听取民主党派、人民团体和各界人士的意见建议，主动接受社会监督、舆论监督。

　　坚持科学理政。加强学习型政府建设，各级政府工作人员要树立终身学习的理念，不断提高理论水平、政策水平，强化产业发展、科技创新、城市建设和社会管理等专业知识的学习，进一步提升引领和推动发展的能力。大兴调查研究之风，深入基层问政问计，使各项政策措施更加符合温州实际、符合群众利益。加强制度供给，创新政府管理理念和方式，提高为市场主体服务的水平。实施“互联网+”政务管理新模式，加快构建综合高效的政府服务平台，推动政府管理向科学化、精细化、智慧化和效能化转变。

　　坚持高效施政。大力弘扬敢于担当、勇于破难的精神，严格实行目标管理责任制，对年度重点任务定责任、定时限、定要求，全力推动政务工作提速、提质、提效，切实提高政府执行力。健全常态化督查机制，强化对重要决策、重点工作和重大项目的跟踪督办，推动各项决策部署落到实处。完善绩效管理体系，突出精准考核，既要考核目标结果，又要考核形象进度，充分激发各级干部干事创业的主动性和创造性。加强效能评价和行政问责，持续治理“庸懒散慢”，坚决打通“中梗阻”，确保政令畅通。

　　坚持廉洁从政。深入践行“三严三实”，始终把纪律和规矩挺在前面，严格执行中央八项规定，持之以恒整治“四风”。进一步规范政府采购、工程招投标和政府投资行为，健全国有资产、公共资金、公共资源监管制度，加强行政监察和审计监督，真正“把权力关进制度的笼子里”。严格落实党风廉政建设“两个主体”责任，深入贯彻《中国共产党廉洁自律准则》和《中国共产党纪律处分条例》，始终保持惩治腐败的高压态势，营造风清气正的政务环境。

　　各位代表！美好蓝图已经绘就，新的征程全面开启。让我们紧密团结在以习近平同志为总书记的党中央周围，在省委、省政府和中共温州市委的坚强领导下，紧紧依靠全市人民，解放思想，开拓创新，扎实工作，为实现“十三五”发展良好开局、建设迈入全面小康社会标杆城市而努力奋斗！
